# Efficacy and Safety of Direct-Acting Antivirals in Elderly Patients with Chronic Hepatitis C: A Nationwide Real-Life, Observational, Multicenter Study from Turkey

**DOI:** 10.5152/tjg.2022.21271

**Published:** 2022-10-01

**Authors:** Yusuf Önlen, Tayibe Bal, Mehmet Çabalak, Nefise Çuvalcı Öztoprak, Nagehan Didem Sarı, Behice Kurtaran, Ebubekir Şenateş, Alper Gündüz, Esra Zerdali, Hasan Karsen, Ayşe Batırel, Rıdvan Karaali, Rahmet Güner, Tansu Yamazhan, Şükran Köse, Nurettin Erben, Nevin İnce, İftihar Köksal, Figen Sarıgül Yıldırım, Gülşen Yörük, Süheyla Kömür, Sibel Kaya, Şaban Esen, Özgür Günal, İlknur Esen Yıldız, Dilara İnan, Şener Barut, Mustafa Namıduru, Selma Tosun, Kamuran Türker, Alper Şener, Kenan Hızel, Nurcan Baykam, Fazilet Duygu, Esragül Akıncı, Güray Can, Ülkü User, Hanefi Cem Gül, Ayhan Akbulut, Güven Çelebi, Mahmut Sünnetçioğlu, Oğuz Karabay, Hayat Kumbasar Karaosmanoğlu, Fatma Sırmatel, Fehmi Tabak

**Affiliations:** 1 Mustafa Kemal University Faculty of Medicine, Antakya, Turkey; 2 Antalya Training and Research Hospital, Antalya, Turkey; 3 İstanbul Training and Research Hospital, İstanbul, Turkey; 4 Çukurova University Faculty of Medicine, Adana, Turkey; 5 Department of Gastroenterology, Medeniyet University Faculty of Medicine, İstanbul, Turkey; 6 Department of Infectious Diseases, Şişli Hamidiye Etfal Training and Research Hospital, İstanbul, Turkey; 7 Department of Infectious Diseases, Haseki Training and Research Hospital, İstanbul, Turkey; 8 Department of Infectious Diseases and Clinical Microbiology, Harran University Faculty of Medicine, Urfa, Turkey; 9 Department of Infectious Diseases, Kartal Training and Research Hospital, İstanbul, Turkey; 10 Department of Infectious Diseases and Clinical Microbiology, Tekirdağ Namık Kemal University Faculty of Medicine, Tekirdağ, Turkey; 11 Department of Infectious Diseases and Clinical Microbiology, Ankara City Hospital, Ankara, Turkey; 12 Department of Infectious Diseases and Clinical Microbiology, Ege University Faculty of Medicine, İzmir, Turkey; 13 Department of Infectious Diseases, Tepecik Training and Research Hospital, İzmir, Turkey; 14 Department of Infectious Diseases and Clinical Microbiology, Eskişehir Osman Gazi University, Eskişehir, Turkey; 15 Department of Infectious Diseases and Clinical Microbiology, Düzce University Faculty of Medicine, Düzce, Turkey; 16 Department of Infectious Diseases and Clinical Microbiology, Karadeniz Technical University Faculty of Medicine, Trabzon, Turkey; 17 Department of Infectious Diseases, Antalya Training and Research Hospital, Antalya, Turkey; 18 Department of Infectious Diseases, İstanbul Training and Research Hospital, İstanbul, Turkey; 19 Department of Infectious Diseases and Clinical Microbiology, Çukurova University Faculty of Medicine, Adana, Turkey; 20 Department of Infectious Diseases and Clinical Microbiology, İstanbul University-Cerrahpaşa, Cerrahpaşa Faculty of Medicine, İstanbul, Turkey; 21 Department of Infectious Diseases and Clinical Microbiology, Samsun 19 Mayıs University Faculty of Medicine, Samsun, Turkey; 22 Department of Infectious Diseases, Samsun Training and Research Hospital, Samsun, Turkey; 23 Department of Infectious Diseases and Clinical Microbiology, RTE University Faculty of Medicine, Rize, Turkey; 24 Department of Infectious Diseases and Clinical Microbiology, Akdeniz University Faculty of Medicine, Antalya, Turkey; 25 Department of Infectious Diseases and Clinical Microbiology, Gaziosmanpaşa University Faculty of Medicine, Tokat, Turkey; 26 Department of Infectious Diseases and Clinical Microbiology, Gaziantep University Faculty of Medicine, Gaziantep, Turkey; 27 Department of Infectious Diseases, Bozyaka Training and Research Hospital, İzmir, Turkey; 28 Department of Infectious Diseases, Bağcılar Training and Research Hospital, İstanbul, Turkey; 29 Department of Infectious Diseases and Clinical Microbiology, Çanakkale 18 Mart University Faculty of Medicine, Çanakkale, Turkey; 30 Department of Infectious Diseases and Clinical Microbiology, Gazi University Faculty of Medicine, Ankara, Turkey; 31 Department of Infectious Diseases and Clinical Microbiology, Hitit University Faculty of Medicine, Çorum, Turkey; 32 Department of Infectious Diseases, Ankara Dr. Abdurrahman Yurtaslan Oncology Training and Research Hospital, Ankara, Turkey; 33 Department of Infectious Diseases, Ankara Numune Training and Research Hospital, Ankara, Turkey; 34 Department of Gastroenterology, Bolu İzzet Baysal University Faculty of Medicine, Bolu, Turkey; 35 Department of Infectious Diseases, Gülhane Training and Research Hospital, Ankara, Turkey; 36 Department of Infectious Diseases and Clinical Microbiology, Fırat University Faculty of Medicine, Elazığ, Turkey; 37 Department of Infectious Diseases and Clinical Microbiology, Bülent Ecevit University Faculty of Medicine, Zonguldak, Turkey; 38 Department of Infectious Diseases and Clinical Microbiology, Yüzüncü Yıl University Faculty of Medicine, Van, Turkey; 39 Department of Infectious Diseases and Clinical Microbiology, Sakarya University Faculty of Medicine, Hatay, Turkey; 40 Department of Infectious Diseases, Bakırköy Dr. Sadi Konuk Training and Research Hospital, İstanbul, Turkey; 41 Department of Infectious Diseases and Clinical Microbiology, Bolu İzzet Baysal University Faculty of Medicine, Bolu, Turkey; 42 İstanbul University-Cerrahpaşa, Cerrahpaşa Medical Faculty of Medicine, İstanbul, Turkey

**Keywords:** Age, chronic hepatitis C, direct-acting antiviral agents, elderly, Turkey

## Abstract

**Background:**

The number and proportion of elderly patients living with chronic hepatitis C are expected to increase in the coming years. We aimed to compare the real-world efficacy and safety of direct-acting antiviral treatment in elderly and younger Turkish adults infected with chronic hepatitis C.

**Methods:**

In this multicenter prospective study, 2629 eligible chronic hepatitis C patients treated with direct-acting antivirals between April 2017 and December 2019 from 37 Turkish referral centers were divided into 2 age groups: elderly (≥65 years) and younger adults (<65 years) and their safety was compared between 2 groups in evaluable population. Then, by matching the 2 age groups for demographics and pretreatment risk factors for a non-sustained virological response, a total of 1516 patients (758 in each group) and 1244 patients (622 in each group) from the modified evaluable population and per-protocol population were included in the efficacy analysis and the efficacy was compared between age groups.

**Results:**

The sustained virological response in the chronic hepatitis C patients was not affected by the age and the presence of cirrhosis both in the modified evaluable population and per-protocol population (*P* = .879, *P* = .508 for modified evaluable population and *P* = .058, *P* = .788 for per-protocol population, respectively). The results of the per-protocol analysis revealed that male gender, patients who had a prior history of hepatocellular carcinoma, patients infected with non-genotype 1 hepatitis C virus, and patients treated with sofosbuvir + ribavirin had a significantly lower sustained virological response 12 rates (*P* < .001, *P *= .047, *P* = .013, and *P* = .025, respectively).

**Conclusion:**

Direct-acting antivirals can be safely used to treat Turkish elderly chronic hepatitis C patients with similar favorable efficacy and safety as that in younger adults.

## Introduction

According to the World Health Organization (WHO) report, there were an estimated 71 million people (1% of the world population) who were living with chronic hepatitis C (CHC) infection, and 1.75 million new hepatitis C virus (HCV) infections occurred worldwide.^1^ Hepatitis C virus infection prevalence and secondary liver diseases are shown to be associated with advanced age.^2^ However, elderly patients were less likely to receive recommended therapies in the past because of toxicity and poor efficacy with interferon (IFN)-based therapies.^3,4^

With the advances in HCV treatment with direct-acting antivirals (DAAs), HCV infection has become curable even in difficult-to-treat patient groups,^5^ which might enable the elimination of HCV infection, although there is no effective vaccine against the HCV virus.^6,7^ However, because elderly cases (≥60 years of age) constitute a significant portion (40%-75%) of the burden of CHC, increasing our knowledge about the efficacy and safety of DAAs in these patient groups is crucial.^8^

The cure of HCV with DAAs in elderly patients has been shown to be associated with a decreased incidence of liver-related events and liver-related mortality which was similar to those shown in younger patients.^9^ Furthermore, observational data from different nations’ real-life cohorts revealed that DAA treatment is effective and safe for the elderly.^10–12^ As is known, Turkey is located in the Eastern Mediterranean Region, which has reported the highest HCV prevalence in the world, yet little is known about the efficacy and safety of DAAs in the elderly Turkish population.^1,13,14^

The objective of this prospective, multicenter study is to evaluate whether DAA treatment is as effective and safe for elderly Turkish patients as for younger adults.

## MATERIALS and Methods

This nationwide, multicenter prospective, non-randomized observational study conducted by the Viral Hepatitis Society and Infectious Diseases and Clinical Microbiology Specialty Society of Turkey, including 2629 CHC patients, administered a DAA regimen to patients from 37 Turkish referral centers between April 2017 and December 2019. Patients with any prior IFN-free DAA treatment and patients aged <18 years were excluded from this study. The demographical, clinical, and laboratory data were collected via a web-based reporting system.

The participants were divided into 2 age groups: elderly (≥65 years) and younger adults (<65 years). The elderly group was further divided into 3 subgroups: 65-74 years old and ≥75 years old. Efficacy and safety were compared between 2 age groups and also subgroups of the elderly group.

Patients received 1 of the 3 DAA regimens: ledipasvir (LDV) + sofosbuvir (SOF) ± ribavirin (RBV), ombitasvir (OBV) + paritaprevir/ritonavir (PTV/r) ± dasabuvir (DSV) ± RBV, and SOF + RBV. The RBV dose was initiated based on the body weight of patients. The choice of DAA regimen and all other therapeutic decisions were at the discretion of the treating physician based on current guideline recommendations and insurance coverage.

To avoid undesirable side effects and bias related to drug–drug interactions, all medications used by the patients were controlled from online drug interactions databases.

Laboratory tests including HCV RNA level, complete blood count, serum alanine aminotransferase, aspartate aminotransferase, total bilirubin, kreatinin, international normalized ratio, albumin, and alpha-fetoprotein were obtained at baseline, week 4, end of the treatment, and 12 weeks of post-treatment follow-up. Hepatitis C virus genotyping was performed only at baseline. Adherence to the DAA regimen and reported adverse events were recorded at each clinical visit.

Hepatitis C virus genotype was determined by reverse transcriptase-polymerase chain reaction (RT-PCR) with genotype-specific primers, and plasma HCV RNA levels were determined by a quantitative real-time PCR-based method routinely available in each participating center.

Serious adverse events possibly related to the treatment regimen were reported to national regulatory/public health authorities.

Elderly patients were defined in accordance with the WHO definition.^15^ Patients were identified as having cirrhosis if they had a liver biopsy showing cirrhosis (Metavir F4 or Ishak 5-6)^16^ or an ultrasound report suggesting cirrhosis (e.g., blunt liver edge, irregular surface, splenomegaly, dilated portal vein, and hypertrophic left lobe) or clinical features of cirrhosis (e.g., ascites, esophageal, or gastric varices). Cirrhotic patients with a history of variceal bleeding, ascites, or hepatic encephalopathy were defined as having decompensated cirrhosis.

The primary outcome was the proportion of patients achieving a sustained virological response (SVR), which is defined as an undetectable HCV viral load 12 weeks after the completion of treatment. The secondary outcomes identified predictors of non-SVR in the elderly group, reported adverse events (AEs) including severe adverse events (SAEs) and death, and also reported the rate of RBV dose modification/discontinuation.

Effectiveness assessments other than SVR12 included early virological response (undetectable serum HCV RNA at the end of the fourth week of the treatment), virologic breakthrough (detectable HCV RNA during the treatment when previously undetectable), and relapse (detectable HCV RNA after treatment when previously undetectable at the end of treatment).

All patients who received at least 1 dose of the DAA regimen were evaluated for safety, and serious AEs were defined based on National Cancer Institute Common Terminology Criteria for Adverse Events Version 4.03.^17^ Hepatic decompensation was considered as a liver-related event, not an SAE.

The study was approved by the Ethics Committee of İstanbul University Cerrahpaşa - Cerrahpaşa Faculty of Medicine University Medical Faculty. Before the beginning of the study, written informed content was obtained from all participants. This study was registered at clinicaltrials.gov, with registration number NCT03145844.

### Statistical Analysis

Effectiveness analysis was performed with both modified evaluable population (mEP) and per-protocol (PP) populations. The mEP population included all patients who had at least 1 post-baseline measurement for the primary outcome, while the PP population included all patients who adhered to the study protocol (completed duration of treatment and had available data for primary outcome) and those who discontinued treatment due to (S)AEs but excluded those who discontinued treatment for reasons other than (S)AEs or were lost to follow-up. All patients who received at least 1 dose of the DAA regimen (evaluable population [EP] population) were evaluated for safety.

The Statistical Package for Social Sciences version 23.0 software (IBM Corp.; Armonk, NY, USA) was used for statistical analysis. Continuous variables were expressed as median and interquartile range and compared with Mann–Whitney *U* test or Kruskal–Wallis test, whereas categorical variables were expressed as count and percentages and compared with chi-square or Fisher’s exact test when appropriate. A logistic regression analysis was performed to determine independent risk factors associated with treatment failure in the mEP and PP populations. However, we do not report their results here. Potential confounders for the multivariate model were selected based on literature and the significance of univariate analysis. Statistical significance was defined by a *P*-value of less than .05.

### Propensity Score Matching Analysis

To reduce the effects of possible confounding factors and eliminate selection bias, a propensity score matching analysis was performed by matching ≥65 years and <65 years groups to select patients with similar baseline characteristics in mEP and PP populations. After estimation of the propensity scores based on potential confounders, ≥65 years patients were propensity score matched using a simple 1 : 1 nearest-neighbor matching without replacement to <65 years patients. We used a caliper of width equal to 0.2 of the standard deviation of the logit of the propensity score. Standardized differences were calculated to assess group balances before and after the weights with imbalance being defined as an absolute value greater than 0.20 (small effect size). R program (version 4.1.1 for Mac) was used for statistical analysis.

## Results

### Characteristics of Patients

During the study period, 2713 patients underwent treatment with a DAA regimen. Of those, 2629 patients who fulfilled inclusion criteria were included in the study.

**Figure 1.**
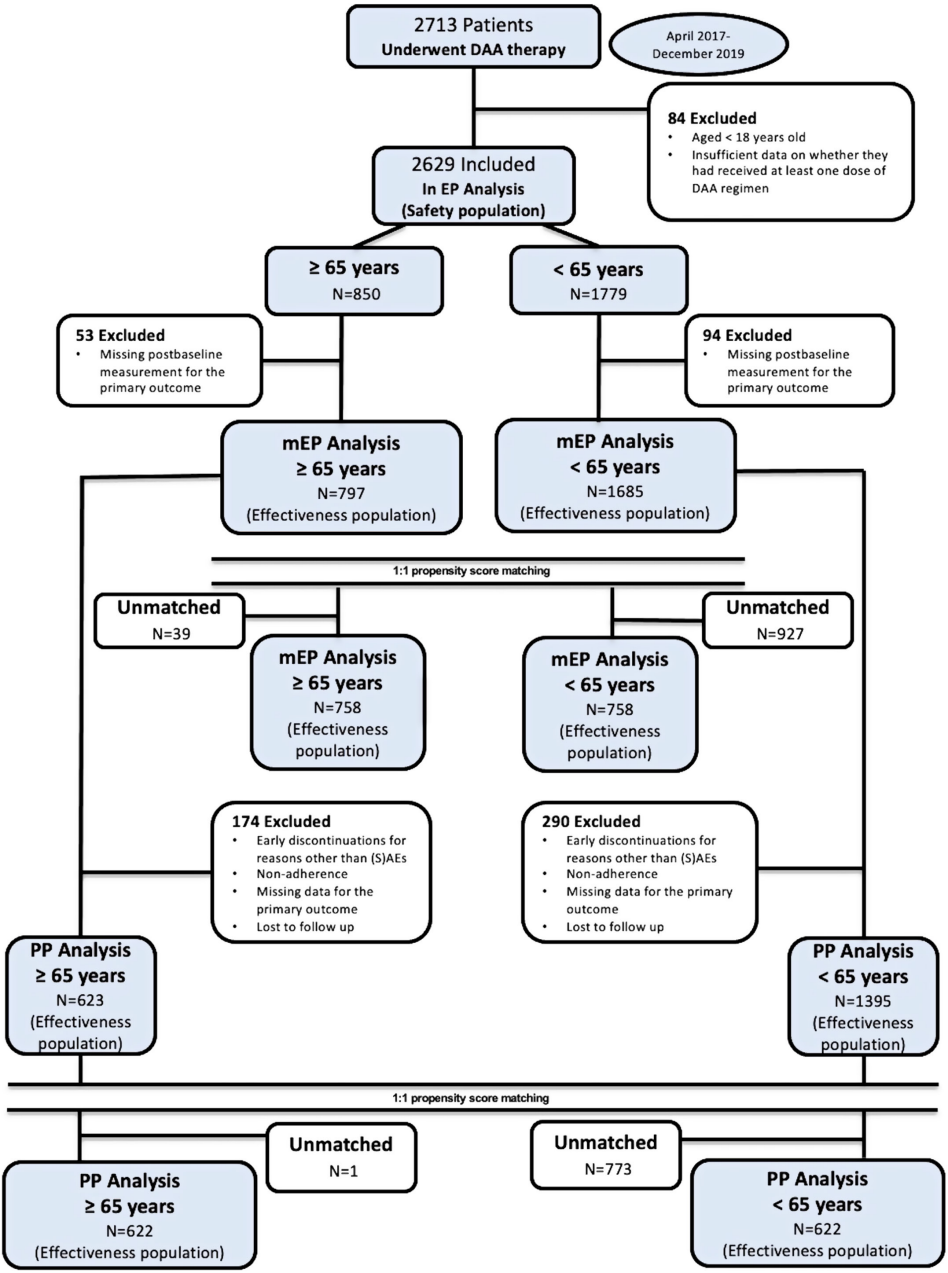
Flowchart showing the selection of the study populations. EP, evaluable population; mEP, modified evaluable population; PP, per-protocol populations.

Our unmatched initial cohort included 2629 patients, 850 (32.3%) were ≥65 years of age, of whom 197 (23.1%) patients were ≥75 years, and 1779 (67.7%) were <65 years of age. By matching the 2 age groups for demographics and pretreatment risk factors for non-SVR, a total of 1516 patients (758 in each group) and 1244 patients (622 in each group) from mEP and PP populations were included in the outcome analysis. The study flowchart is provided in Figure 1.

Table 1 presents the comparison of demographics and baseline clinical characteristics of the age groups before and after matching in mEP and PP populations. The higher proportion of comorbidity, treatment experience, cirrhosis, genotype 1 (GT1) infection, hepatocellular carcinoma (HCC), and female gender in the ≥65 years group compared to <65 years group seen in the unmatched mEP and PP populations were no longer significant after matching (Table 1).

**Figure 2.**
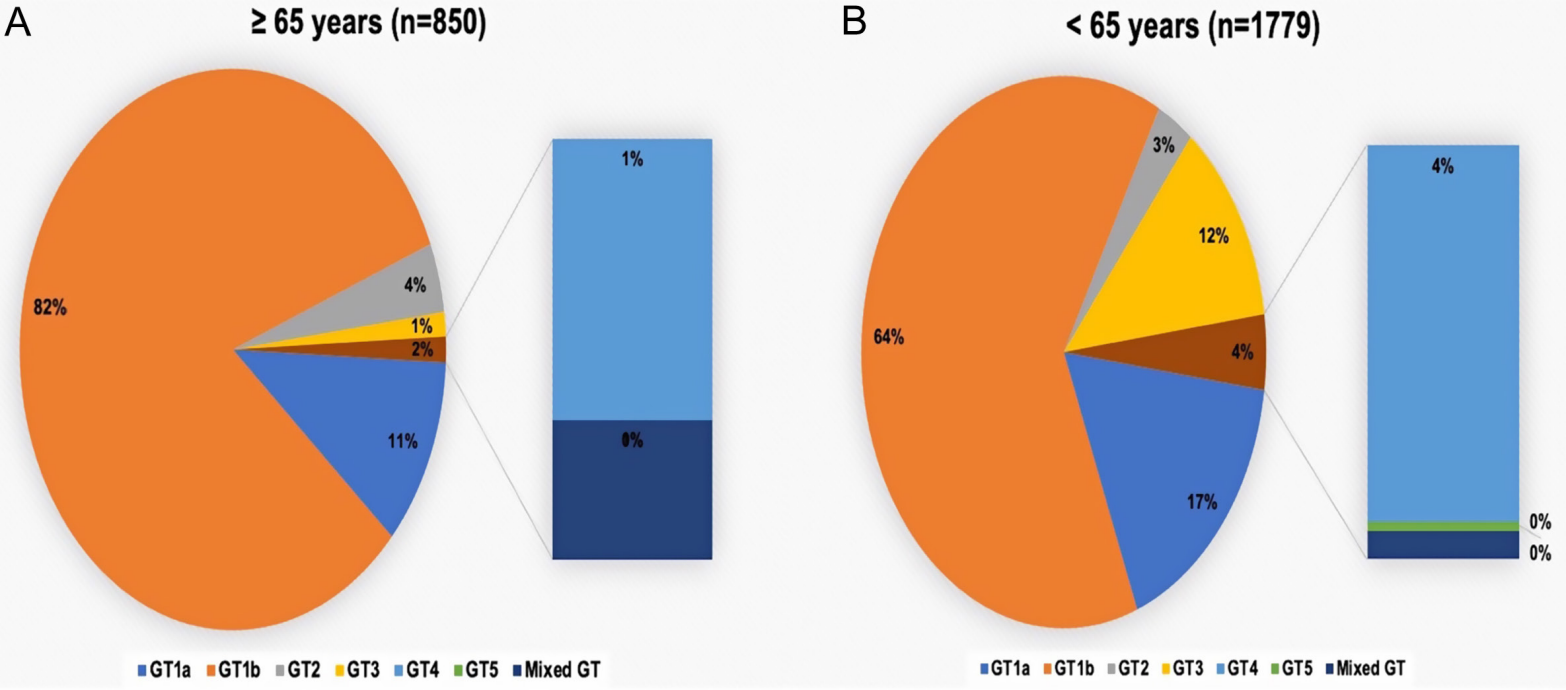
Genotype distribution according to age groups: ≥65 years (A) versus <65 years (B).

The ≥65 years group had a significantly higher proportion of patients infected with HCV genotype 1, while infection with genotype 2 was more frequent in younger adults in the PP population (Figure 2).

The details of the 3 DAA regimens received and the usage frequency of RBV in the 2 age groups are presented in Table 2. The majority of the patients received paritaprevir, ritonavir, ombitasvir, and dasabuvir (PrOD)-based regimen in the elderly group as well as in younger ones (53.9% and 70.3%, respectively). On the contrary, the rate and also the number of patients receiving LDV/SOF **±** RBV combination in the <65 years group were quite low (27.7% [172/622]) as compared with the ≥65 years group (41.8% [260/622]).

### Effectiveness Analysis

A total of 1516 (758 in each age group) and 1244 (622 in each age group) patients in the mEP and PP populations were included for treatment outcome analysis, respectively.

Both in the unmatched and matched populations, 2 age groups showed similar SVR rates (*P* = .938 and *P* = .897 for mEP, *P* = .577 and *P* = .058 for PP population, respectively) (Table 3). Likewise, patients ≥75 years of age had similar SVR rates compared to those of 65-74 years of age among the unmatched ≥65 years group (*P* = 1.000, 99.2% vs 98.8%, respectively, in the PP analysis and *P* = .06, 82.9% vs 88.1% in the mEP analysis).

**Figure 3.**
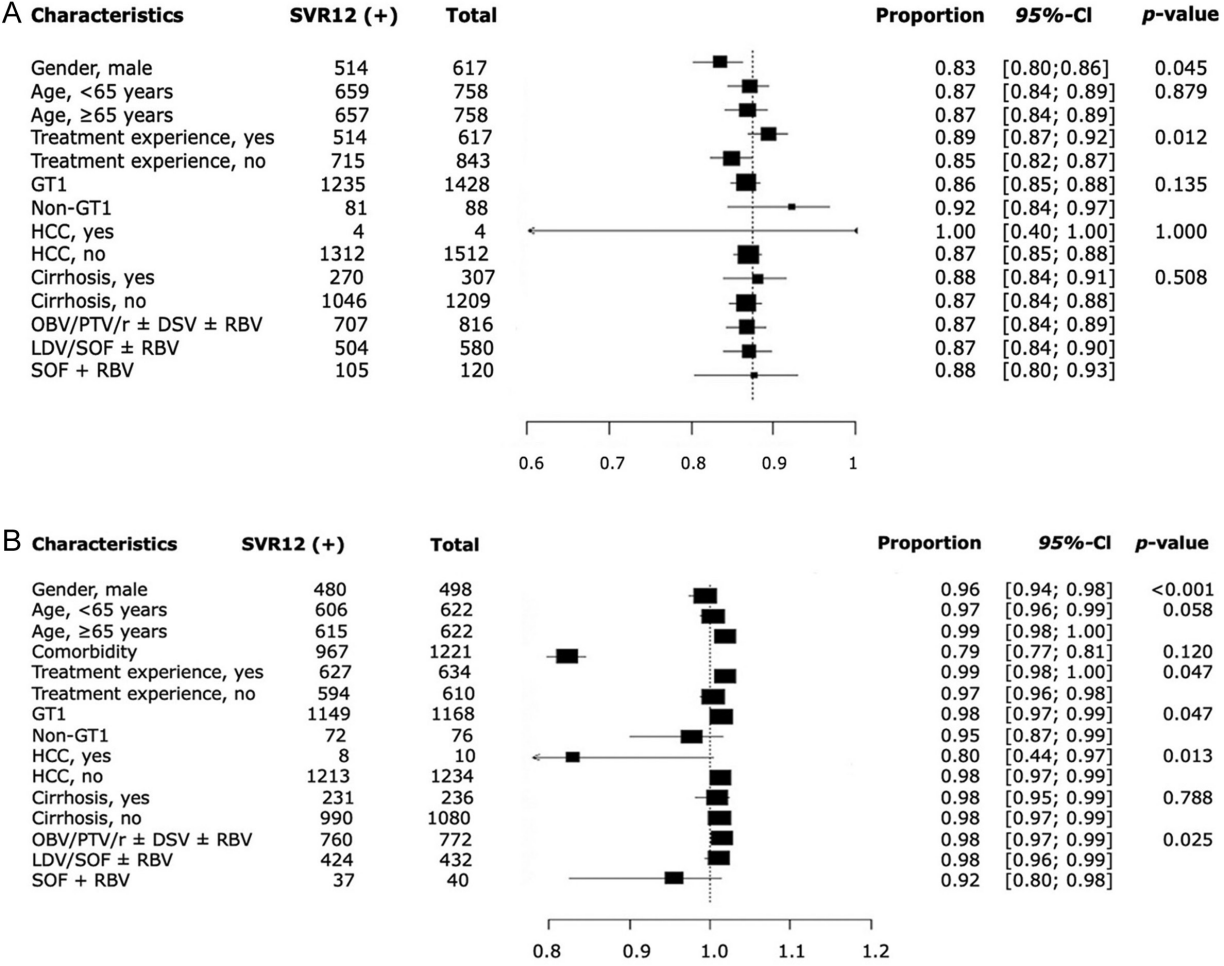
A. Rates of SVR12 according to the different characteristics in the modified evaluable (mEP) population. GT1, genotype 1; HCC, hepatocellular carcinoma; OBV, ombitasvir; PTV/r, paritaprevir/ritonavir; DSV, dasabuvir; RBV, ribavirin; LDV, ledipasvir; SOF, sofosbuvir. B. Rates of SVR12 according to the different characteristics in the per-protocol (PP) population. GT1, genotype 1; HCC, hepatocellular carcinoma; OBV, ombitasvir; PTV/r, paritaprevir/ritonavir; DSV, dasabuvir; RBV, ribavirin; LDV, ledipasvir; SOF, sofosbuvir.

Figure 3A and B shows SVR12 rates stratified by potential confounders in mEP analysis and in PP analysis, respectively. Additionally, matched age groups were compared in terms of SVR12 status according to these variables (Forrest plot in Figure 3A and B). Sustained virological response12 rates of CHC patients were similar between different DAA regimens (for OBV/PTV/r + DSV, LDV/SOF, and SOF + RBV, respectively) both in mEP analysis (87%, 87%, and 88%, respectively) and PP analysis (98%, 98%, and 92%, respectively). The SVR12 (+) group in the mEP population had a significantly higher proportion of treatment experience, while the male gender was significantly more frequent in the non-SVR group (*P* = .012 and *P* = .045, respectively) (Figure 3A).

The results of the PP analysis revealed that male gender, patients who had a prior history of HCC, patients infected with non-GT1 HCV, and patients treated with SOF + RBV had a lower SVR12 rates (*P* < .001, *P* = .047, *P* = .013, and *P* = .025, respectively) (Figure 3B).

The SVR in the CHC patients was not affected by age and the presence of cirrhosis both in the mEP and PP populations (*P* = .879 and *P* = .508 for mEP population and *P* = .058 and *P* = .788 for PP analysis, respectively) (Figure 3A and B).

### Safety Analysis

A total of 2629 patients in the EP were included for safety analysis. Even though the elderly patients had significantly higher rates of AEs than younger adults (20.1% vs 13.6%, *P *< .001 in EP analysis), AEs were generally mild and occurred at low rates even in patients ≥75 years of age (16.8% [33/196]).

The most common AEs in the elderly group were fatigue, anemia, and pruritus (9.3%, 9.1%, and 7.9%, respectively). Fatigue and pruritus were more common in the elderly group, whereas headache was more common in the younger adults group (Table 4). AEs reported by ≥2% of the patients for both age groups are shown in Table 4.

Severe AEs leading to treatment discontinuation were slightly more common in elderly patients than in younger adults (0.35% [3/850] vs [7/1779] 0.39%, respectively) (Table 4). Nevertheless, the frequency of discontinuation of DAAs due to SAEs was low even in patients aged ≥75 years old (1% [2/196]). A complete list of serious AEs is demonstrated in Table 5.

The elderly patients had a relatively higher mortality rate than young adults (1.65% [14/850] vs 0.39% [7/1779]). Interestingly, the mortality in the elderly was mostly due to comorbidities (64.2% [9/14]) but partly due to liver-related events (21.4% [3/14]). Nevertheless, mortality rates due to liver-related events in the younger adults group were similar to those due to coexisting comorbidities (0.16% [3/1779] vs 0.22% [4/1779]).

In the elderly group, 3 patients died due to liver-related event (3 hepatic decompensation), while 2 patients died due to non-liver-related events (1 acute renal failure and 1 necrotizing fasciitis). Among the younger adults group, 3 patients died due to liver-related events (2 newly detected hepatocellular carcinomas and 1 hepatic decompensation). No SAE-related death was observed both in older and younger adults groups.

Of the 4 patients in the elderly group who died or experienced treatment discontinuation due to hepatic decompensation, 3 received PrOD regimen and 1 received LDV/SOF regimen. On the other hand, only 1 patient who received the PrOD regimen experienced hepatic decompensation in the younger adults group.

In the current study, AEs were significantly higher in patients receiving RBV-containing DAA regimens compared to those receiving DAA regimens without RBV (25.9% [41/158] vs 18.8% [129/688], respectively, *P* = .042).

The use of RBV-containing regimens was more frequent in younger adults than in elderly patients (36.6% and 18.7%, respectively). However, the rates of RBV dose reduction/discontinuation in the elderly were found to be considerably higher than that reported for younger adults (*P* = .036, 6.4% and 4.5%, respectively). Meanwhile, there was no evidence that these higher rates in elderly patients had an influence on reducing their treatment responses (Table 3).

## Discussion

Magnificent progress has been made in the treatment of hepatitis C with the introduction of IFN-free DAAs with extremely high cure rates and minimal side effects in the general population.^8^ There is no reported upper age limit for DAA treatment in the current guidelines.^18^ Despite this, the elderly patients remain a group for which clinicians are hesitant to initiate DAA treatment because of negative past experiences with IFN-based treatments or economic concerns.^9^ Because it is believed that the number and proportion of elderly patients living with hepatitis C infection will increase in the coming years, increasing real-world data on the elderly Turkish population to characterize the efficacy and safety of DAA treatment is essential.^19^ This large nationwide study provides evidence that Turkish elderly CHC patients have similar high SVR rates and approximately identical safety profile to DAAs compared with younger adults.

The SVR in the elderly was not affected by the patient’s age or cirrhosis in the current study. These results are broadly consistent with earlier findings from other cohorts of different countries.^20,21^ However, this finding is contrary to that of Qureshi et al^22^ who found that advancing age negatively affects the HCV treatment outcomes in the case of cirrhosis. This inconsistency may be due to Qureshi et al’s^22^ findings that might be somewhat limited by the small sample size, particularly in the elderly (≥70 years of age) group, and the lack of information on the severity of liver diseases in most of the cases. Since it has been suggested that decompensated cirrhosis may be a risk factor for treatment failure, the low SVR rate observed in their study could be probably due to a high rate of cirrhotic subjects in Child-Turcotte-Pugh-B/C (CTP-B/C) class.^23^

Patients with active HCC were found to be associated with a worse response to DAA treatment compared with those without HCC.^24-26^ Although it is not clear why such a low SVR rate was seen in patients with active HCC, previously, it has been hypothesized that HCV within tumor cells could be relatively inaccessible to DAA agents. It is interesting to note that the present study identified a prior history of HCC as a potential predictor of treatment failure. While this result is consistent with the results of some researchers, others have failed to find a link between a prior HCC history and non-SVR.^26,27^ Because it is well known that prior HCC is associated with increased risk of de novo HCC, we hypothesized that the presence of a possible baseline HCC could not be seen radiologically (maybe in the early stage) in patients with prior HCC might negatively affect SVR.^28^ Hence, caution must be applied when comparing this finding with previously reported studies. Furthermore, well-designed matched control studies with long-term follow-up are warranted to confirm this finding.

Additionally, the male gender was found to be associated with treatment failure in the current study. Since this result has not been found elsewhere, it is difficult to explain this result, but it is probably due to the fact that older men with a long history of HCV infection are more likely to have advanced cirrhosis/liver disease than older women, which can decrease cure rates.^5,23^

In the current study, subgroup analysis stratified by different DAA regimens and potential confounders in non-SVR yielded excellent SVR rates, while SOF + RBV had lower SVR rates than other regimens in the PP population (>97% and 92%, respectively). However, it may not be appropriate to make a definitive conclusion since patients in the 3 treatment arms were not homogeneous. The results of our study are partly in line with the general medical literature, in which SOF + RBV combination treatment is known to have generally low rates of SVR, and this combination is no longer a preferred regimen by The European Association for the Study of the Liver (EASL) and American Association For The Study of Liver Diseases (AASLD).^21,29,30^ Thus, it was surprising that we observed excellent SVR rates (92%) with SOF + RBV treatment in the PP population, even though the deficient number of patients (n = 40, 3.2% of the population) in this treatment arm prevented a clear conclusion from this finding. In reviewing the literature, the response rate for this regimen in the elderly is low in Western countries (88.2%) but is higher (97%) in Asian countries.^31,32^ These differences in SVR rates have been attributed to the lower body mass index and predominance of CC IL28B alleles in the Asian population/Asians.^33^ As a result, racial differences could help to explain the high response rates with SOF + RBV observed in the present study, which is supported by a recent study from Turkey.^34^

Direct-acting antivirals were generally well tolerated in the current study, even in patients aged 75 years and older. Additionally, the most common AEs in the elderly were fatigue, anemia, and pruritus in the present study, which corresponded to those previously reported in elderly populations from different countries.^12,21,23^ Surprisingly, the prevalence of AEs in our elderly group was 21%, relatively lower than the rates reported in previous studies which range from 24% up to 95%.^11,23,35^ This lower percentage of AEs in the present study may reflect differences in study protocols, treatment populations, or covariate adjustment, and it will require further studies. However, not surprisingly, we found that the frequency of AEs was higher when DAA regimens were combined with RBV compared with DAAs alone, which is supported by previous reports.^5,12,36^ Although the use of RBV-containing regimens was more frequent in younger adults than in elderly patients (36.6% and 18.7%, respectively), the incidences of RBV dose reduction/discontinuation in the elderly were found to be considerably higher than those in younger adults. However, this did not adversely affect the SVR, which is consistent with earlier observations.^9^ Based on these findings, the use of RBV should be avoided in elderly patients because of the increased risk of AEs such as anemia. Similar to the results of Dultz et al,^37^ we found that headache was more common among younger adults than elderly patients. However, according to another study, similar rates of headache in elderly patients in comparison to their younger counterparts were founded.^38^ These higher rates of headache in younger adults could be attributed to higher rates of the use of RBV-containing regimens in younger adults than elderly patients in the current study. Indeed, in a study by Bräu et al,^39^ they attributed headaches to RBV-based regimens.

In the present study, although the incidence of serious AEs increased with age, premature discontinuation of DAAs or death due to SAEs was uncommon (less than 1%) even in those ≥75 years of age. These results are similar to those obtained by Dultz et al^21^ while the incidences observed in the current study are far below those observed by Lens et al^40^ who have suggested that advanced age (≥75 years of age) and liver cirrhosis are associated with an increased incidence of SAEs-related premature discontinuation. Consequently, it is possible that our results may have been influenced by the low proportion of patients with advanced age and cirrhosis in the present study.

Another point worth mentioning is that in the current study, we accepted newly diagnosed hepatic decompensation as a liver-related event, not as an SAE. Even though hepatic decompensation during DAA treatment is classified and reported as an SAE in the vast majority of previous studies, recent findings from several studies suggest that this liver-related event might be due to the natural course of advanced cirrhosis rather than being directly related to DAA treatment.^41-43^ This view is also supported by the fact that elderly patients with CHC cases are at a high risk of developing end-stage liver disease even after eradication of HCV, because the regeneration of the liver is slow and usually incomplete following a liver injury.^44,45^ Recently, a meta-analysis including more than 3400 patients (70% of these had cirrhosis) reported that the rates of hepatic decompensation in patients undergoing treatment with a DAA regimen are lower than the estimated rates of annual hepatic decompensation in patients with compensated cirrhosis (0.96% vs 5%-7%) that never received DAA treatment.^46^ Thus, there are still unanswered questions about the safety of DAAs, especially in patients with cirrhosis, upon which future investigations should focus.

Elderly patients had a higher all-cause mortality rate compared with younger patients. This high rate could, however, mainly be attributed to non-liver-related comorbidities. These results are consistent with those of other studies and support the idea that DAA treatment reduces liver-related events and related mortality in elderly as well as in younger adults.^9,40^

The major limitation of this study was that non-homogeneous distributions exist among treatment agents, which limited our ability to compare the efficacy and safety of different DAA regimens. On the other hand, the prospective nature and multicenter setting were the major strengths of our study.

The results of the current study suggested that DAA regimens can be safely used to treat Turkish elderly CHC patients with similar favorable efficacy as that in younger adults, even for those aged 75 years and over. Elderly patients with a prior history of HCC and of the male gender, however, are at higher risk for treatment failure. Nevertheless, future research is needed to address the causal nature of these relations.

## Figures and Tables

**Table 1.** Baseline Demographics and Clinical Characteristics Before and After Propensity Score Matching of the Age Groups in Modified Evaluable Population (mEP) and Per-Protocol (PP) Population

**Characteristics** **mEP population**	**Before Matching**			**After Matching**		
≥**65 years** **n = 797**	<65 years **n = 1685**	*P*	≥**65 years** **n = 758**	<65 years **n = 758**	*P*
**PP population**	**n = 623**	**n = 1395**		**n = 622**	**n = 622**	
Gender, male	305 (38.3)	929 (55.2)	<.001	301 (50.4)	306 (50.4)	.834
Cirrhosis	192 (24.1)	190 (11.3)	<.001	154 (50.2)	153 (49.8)	1.000
GT1	751 (94.3)	1372 (81.5)	<.001	714 (50.0)	714 (50.0)	1.000
HCC	9 (1.1)	4 (0.2)	.004	2 (50.0)	2 (50.0)	1.000
Treatment experience	362 (45.5)	576 (34.2)	<.001	333 (49.5)	340 (50.5)	.756
Comorbidity	402 (50.5)	715 (42.4)	.093	384 (50.6)	412 (54.3)	.862
Baseline HCV RNA, ≥800 000 IU/mL	360 (45.2)	765 (45.4)	.968	327 (43.1)	345 (45.5)	.817
Gender, male	237 (38.0)	758 (54.4)	<.001	237 (38.1)	261 (42.0)	.086
Cirrhosis	157 (25.2)	133 (9.5)	<.001	157 (25.2)	79 (12.7)	<.001
GT1	581 (93.4)	1138 (81.6)	<.001	581 (93.4)	587 (94.4)	.554
Comorbidity	493 (79.3)	589 (42.3)	<.001	493 (79.3)	489 (78.6)	.835
Treatment experience	302 (48.6)	526 (37.7)	<.001	302 (48.6)	332 (53.4)	.100
HCC	7 (1.1)	5 (0.4)	.039	7 (1.1)	3 (0.5)	.204
Baseline HCV RNA, ≥ 800 000 IU/mL	312 (50.1)	662 (47.5)	.834	312 (50.1)	274 (44.0)	.089

Data were expressed as n (%).

GT1, genotype 1; HCC, hepatocellular carcinoma; HCV, hepatitis C virus; RNA, ribonucleic acid; mEP, modified evaluable population; PP, per-protocol population.

**Table 2.** DAA Regimens According to Age Groups in PP population

	≥ 65 years (n = 622)	<65 years (n = 622)	**Total (n = 1244)**
OBV/PTV/r ± DSV ± RBV	335 (53.9)	437 (70.3)	772 (62.1)
LDV/SOF ± RBV	260 (41.8)	172 (27.7)	432 (34.7)
**SOF + RBV**	27 (4.3)	13 (2.1)	40 (3.2)
**The usage frequency of RBV **	82 (13.1)	102 (16.3)	184 (14.7)

Data were expressed as n (%).

DSV, dasabuvir; LDV, ledipasvir; OBV, ombitasvir; PTV/r, paritaprevir/ritonavir, RBV, ribavirin; SOF, sofosbuvir.

**Table 3.** Comparison of the SVR12 Rates Before and After Propensity Score Matching According to Age Groups in mEP and PP Populations

**Characteristics**	**mEP Population**			**PP Population**		
**SVR12 (+)**	**Non-SVR**	*P*	**SVR12 (+)**	**Non-SVR**	*P*
**Before matching**			.938			.577
≥**65 years**	692 (86.9)	104 (13.1)		615 (98.8)	7 (1.2)	
<65 years	1465 (87.0)	218 (13.0)		1374 (98.5)	20 (1.5)	
**After matching**			.879			.058
≥**65 years**	657 (86.7)	101 (13.3)		615 (98.8)	7 (1.2)	
<65 years	659 (86.9)	99 (13.1)		606 (97.4)	16 (2.6)	

Data were expressed as n (%).

mEP, modified evaluable population; PP, per-protocol population; SVR, sustained virological response.

**Table 4.** Adverse Events by Age Groups in Evaluable Population (EP)

**Variables**	≥**65 Years (n = 850)**	<65 Years (n = 1779)	*P*
Any AE, n (%)	170 (20.1)	242 (13.6)	<.001
Grade 2 or 3 hyperbilirubinemia	23 (2.7)	33 (1.9)	.156
Significant anemia (Hb <10 g/dL)	77 (9.1)	79 (4.5)	<.001
RBV dose reduction or discontinuation	54 (6.4)	79 (4.5)	.036
Common AEs (≥2%)			
Fatigue	79 (9.3)	117 (6.4)	.008
Pruritus	67 (7.9)	80 (4.5)	<.001
Insomnia	20 (2.4)	51 (2.9)	.450
Headache	21 (2.5)	80 (4.5)	.014
Nausea	32 (3.8)	43 (2.3)	.051

Data were expressed as n (%).

AEs, adverse events; Hb, hemoglobin; RBV, ribavirin.

**Table 5.** Severe Adverse Events According to Age Groups in Evaluable Population (EP)

**Patient ID**	**Age, Years**	**Gender**	**SAE**	**Regimen**	**Time After Treatment Initiation (Weeks)**	**Cirrhosis Status**	**Leading to Treatment Discontinuation**
≥**65 years**							
1	71	F	Exacerbated dyspnea	LDV/SOF	8	Non-cirrhotic	Treatment discontinuation
2	68	F	Angioedema	OBV/PTV/r + DSV	1	Non-cirrhotic	Treatment discontinuation
3	83	M	Severe constipation	OBV/PTV/r + DSV	8	Non-cirrhotic	Treatment discontinuation
<65 years							
1	57	M	Angioedema	OBV/PTV/r + DSV	8	Non-cirrhotic	Treatment discontinuation
2	61	F	Severe urticaria	OBV/PTV/r + DSV	4	CTP-A	Treatment discontinuation
3	45	M	Severe bleeding	SOF + RBV	5	Non-cirrhotic	Treatment discontinuation
4	57	F	Severe constipation	OBV/PTV/r + DSV	4	Non-cirrhotic	Treatment discontinuation
5	62	F	Severe constipation	OBV/PTV/r + DSV	2	Non-cirrhotic	Treatment discontinuation
6	52	F	Severe constipation	OBV/PTV/r + DSV	5	Non-cirrhotic	Treatment discontinuation
7	57	F	Exacerbation of psychosis	LDV/SOF	6	CTP-A	Treatment discontinuation

Data were expressed as n (%).

SAE, severe adverse event; DSV, dasabuvir; LDV, ledipasvir; OBV, ombitasvir; PTV/r, paritaprevir/ritonavir; RBV, ribavirin; SOF, sofosbuvir; CTP, Child–Turcotte–Pugh.
